# Mitochondria Associated Germinal Structures in Spermatogenesis: piRNA Pathway Regulation and Beyond

**DOI:** 10.3390/cells9020399

**Published:** 2020-02-10

**Authors:** Xiaoli Wang, Chunyu Lv, Ying Guo, Shuiqiao Yuan

**Affiliations:** 1 Institute Reproductive Health, Tongji Medical College, Huazhong University of Science and Technology, Wuhan 430030, Hubei, China; wangxiaoli@hust.edu.cn (X.W.); chunyuwendy@163.com (C.L.); 2 Key Laboratory of Male Reproductive Health, National Health Commission of the People’s Republic of China, Beijing 100081, China; guoying0223@163.com; 3 Shenzhen Huazhong University of Science and Technology Research Institute, Shenzhen 518057, Guangdong, China

**Keywords:** nuage, intermitochondrial cement, piP-body, piRNA, mitochondria, spermatogenesis

## Abstract

Multiple specific granular structures are present in the cytoplasm of germ cells, termed nuage, which are electron-dense, non-membranous, close to mitochondria and/or nuclei, variant size yielding to different compartments harboring different components, including intermitochondrial cement (IMC), piP-body, and chromatoid body (CB). Since mitochondria exhibit different morphology and topographical arrangements to accommodate specific needs during spermatogenesis, the distribution of mitochondria-associated nuage is also dynamic. The most relevant nuage structure with mitochondria is IMC, also called pi-body, present in prospermatogonia, spermatogonia, and spermatocytes. IMC is primarily enriched with various Piwi-interacting RNA (piRNA) proteins and mainly functions as piRNA biogenesis, transposon silencing, mRNA translation, and mitochondria fusion. Importantly, our previous work reported that mitochondria-associated ER membranes (MAMs) are abundant in spermatogenic cells and contain many crucial proteins associated with the piRNA pathway. Provocatively, IMC functionally communicates with other nuage structures, such as piP-body, to perform its complex functions in spermatogenesis. Although little is known about the formation of both IMC and MAMs, its distinctive characters have attracted considerable attention. Here, we review the insights gained from studying the structural components of mitochondria-associated germinal structures, including IMC, CB, and MAMs, which are pivotal structures to ensure genome integrity and male fertility. We discuss the roles of the structural components in spermatogenesis and piRNA biogenesis, which provide new insights into mitochondria-associated germinal structures in germ cell development and male reproduction.

## 1. Introduction

Since the advent of electron microscopy, particular attention has been paid to the unique structure in germ cells, termed as nuage, the French word for cloud, initially employed by Andri and Rouiller in 1957. Over 80 animals in eight phyla are reported as having a nuage structure in their germ cells [1]. Nuage are characterized by an amorphous shape, the absence of surrounding membranes, an abundance of RNAs and proteins, and a close association with mitochondria clusters or immediately adjacent to the nuclear envelope of germ cells, including intermitochondrial cement (IMC, also named pi-body), piP-body, and chromatoid body (CB) in mammalian germ cells, according to their localization, morphology, and/or biochemical properties [1,2,3]. 

Mitochondria-associated ER membranes (MAMs) are tight structural contacts, also named as MERCs (mitochondria-ER contacts); ~20% of the mitochondrial surface are jointly opposing and contact directly with the ER, yielding to approximately 10 and 30 nm in distance [4,5]. MAMs participate in several cellular signaling pathways, including calcium transmission, phospholipid exchange, intracellular trafficking, autophagy, ER stress, mitochondrial biogenesis, and inflammasome formation. Disturbances to MAMs lead to neurodegenerative diseases and cancer [6,7]. Our previous work reported that MAMs are abundant in both human and mouse spermatogenic cells, and contain many crucial proteins that are associated with the Piwi-interacting RNA (piRNA) pathway [8]. However, the functions of MAMs are still mysterious in spermatogenesis. 

**Figure 1:**
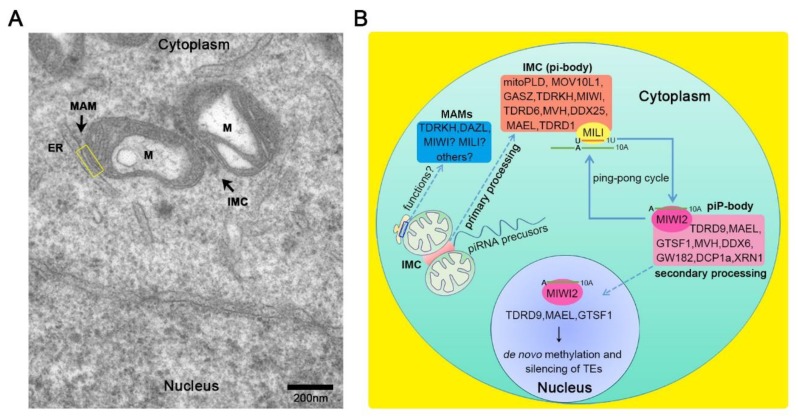
The ultra-structures, components, and functions of intermitochondrial cement (IMC) and mitochondria-associated ER membranes (MAMs) in male germ cells are summarized. (**A**) TEM image showing the morphology of IMC and MAMs (yellow rectangle area) in spermatocytes. M: mitochondria; ER: endoplasmic reticulum. (**B**) The summary of the components and functions of IMC (also called pi-bodies), piP-bodies, and MAMs in Piwi-interacting RNA (piRNA) biogenesis during mouse spermatogenesis. After piRNA precursors are exported from the nucleus, they process by factors located in the IMC into piRNA intermediates, then load to MILI protein and are cleaved, generating mature primary piRNA with 1U signature. The MILI-bound piRNA then engages in the ping-pong cycle leading to the cleavage of complementary antisense transcripts, producing secondary piRNA with 10A signature, and then are loaded to either MILI or MIWI2. Loading of MIWI2 with secondary piRNA induces its translocation into the nucleus where it functions in the methylation and silencing to transposable elements (TEs) together with other nuclear factors. However, the components of MAMs and how MAMs regulate the piRNA pathway still need to be further explored. Non-standard abbreviations in this review are shown: IMC, intermitochondrial cement; CB, chromatoid body; MAMs, mitochondria-associated ER membranes; TEM, transmission electron microscope; MVH, mouse vasa homolog; TDRD1/MTR-1, Tudor domain-containing 1/Mouse Tudor repeat-1; TDRD6, Tudor domain-containing 6; MOV10L1, Moloney leukemia virus 10 like 1; TDRKH, Tudor and KH domain-containing protein.

In this review, we review the insights gained from studying the structural components of mitochondria-associated germinal structures, including IMC, CB, and MAMs, which are pivotal structures to ensure genome integrity and male fertility. We summarize the ultra-structure of IMC and MAMs in mouse spermatocyte, as shown in Figure 1A. We also discuss the roles of the structural components in piRNA biogenesis and propose that further study could unravel new insights into mitochondria-associated germinal structures in spermatogenesis.

## 2. The Concepts and Discoveries of Mitochondria-Associated Germinal Structures 

IMC is clusters formed between mitochondria, apparently “cementing” them together. In mice, IMC presents in gonocytes, spermatogonia, and meiotic spermatocytes until later pachytene spermatocytes and the most known proteins localized in IMC are Tudor domain-containing 1 (TDRD1) and MILI [2,3]. Considering IMC is a primary cytoplasmic localization site of the piRNA pathway proteins, it also named as pi-body. piP-body is another type of nuage that is also close to mitochondria but does not colocalize with IMC. It derived from simultaneously containing both the piRNA pathway and P-body components (control RNA degradation/translational including the miRNA- and small interfering RNA (siRNA)-mediated pathways), such as MIWI2, MAEL, and TDRD9, which are vital piRNA regulating proteins in spermatogenesis enriched in piP-bodies, and DDX6, a P-body component localized in piP-body as well [3]. The chromatoid body (CB) first appears as thick cytoplasmic fibers and granulated material in the interstices of mitochondrial clusters and the perinuclear area of late pachytene spermatocytes. After meiosis, the chromatoid body condenses into one single lobulated, perinuclear granule in round spermatids and disassembles later during spermatid elongation [1,2]. The transcriptome and proteome analysis of the chromatoid body, together with the male infertility phenotypes of chromatoid body protein deleted animal models elucidates that CB is enriched with mRNA, microRNA, piRNA, and RNA binding proteins, thereby regulating mRNA storage, nonsense-mediated RNA decay (NMD) pathway, and piRNA pathway [9,10]. In contrast, IMC is much less informative, although it is persistently present in male and female germ cells. Since the presence of piRNA pathway components in IMC, increasing research has emerged in recent years. 

Importantly, there is another structure called mitochondria-associated ER membranes (MAMs), which are also abundantly present in the cytoplasm of spermatogonia, spermatocytes, and round spermatids, and enriched with many critical proteins in piRNA pathways such as MIWI, MILI, GASZ, and Tudor and KH domain-containing protein (TDRKH) [8]. Although MAMs attract much attention and are deeply investigated in neurodegenerative diseases and cancer, there is much less research on the functions of MAMs in spermatogenesis. However, the essential involvement of MAM proteins in spermatogenesis was identified in both mouse and human models; an example is TDRKH, which participates in primary piRNA biogenesis in the germline [8,11]. 

Although the molecular functions of the nuage are enigmatic, accumulating research in the past few years discloses its accommodation of copious components responsible for piRNA biogenesis and metabolism pathway to silence transposable elements (TE). Here, we draw on recent discoveries to summarize the current understanding of these mitochondria-associated unique structures in mouse germ cells, including IMC, CB, and MAMs. 

## 3. The Components and Functions of IMC and CB in Spermatogenesis and piRNA Biogenesis 

Based on present research, the proteins enriched in IMC are mainly Tudor domain family and Piwi proteins. The name of the Tudor domain family obtained from the *Drosophila melanogaster Tudor (Tud) gene*, a maternal effect grandchildless mutant found in 1985, is essential during oogenesis for the formation of primordial germ cells (pole cells) and normal embryonic abdominal segmentation [12]. The Tudor domain also belongs to the “royal family” of modules that specifically recognize arginine- or lysine-methylated ligands to facilitate protein–protein interaction [13]. Tudor proteins play a multiplicity of cellular functions, including RNA metabolism, germ cell development, transposon silencing, DNA damage response, histone modification, and chromatin remolding [14]. In recent years, a collection of Tudor proteins were validated as primarily expressed in germ cells, where they interact with arginine-methylated P-element-induced wimpy testis (PIWI) proteins to adjust the biogenesis and usage of piRNA to silence transposable elements in spermatogenesis, such as TDRD1 and TDRD6 [15,16]. 

PIWI (P-element-induced wimpy testis) proteins are an evolutionarily conserved subclade of the Argonaute (AGO) family, which primarily express in mammalian germ cells and are essential for germ cell development, stem cell self-renewal, epigenetic regulation, and transposon silencing [17,18]. PIWI proteins specifically associate with piRNAs (Piwi-interacting RNAs), which are a class of small RNAs, commonly 24~32 nucleotides (nt) in length. piRNAs join and associate with PIWI proteins and guide them to select the target sequence to protect the genome integrity from transposon activation mainly. There are three PIWI proteins in mice, including MILI (PIWIL2), MIWI2 (PIWIL4), and MIWI (PIWIL1), and all of them play pivotal and non-redundant roles in spermatogenesis [19,20,21]. Each of them shows a unique expression pattern, but all three proteins enriched at the nuage of male germ cells. MILI expressed in the cytoplasm granules both in fetal primordial germ cells (PGCs) and postnatal spermatocytes until spermatids (E12.5 to adult). MIWI2 displays a relatively narrow expression window from ~ E14.5 to P3, and is localized in the cytoplasm and nucleus [3,21]; MIWI2 also appears as granules in the cytoplasm, but only very few can co-stain with MIWI-positive granules, owing to different granule distribution that MILI, mainly in IMC, and MIWI2 in piP-body, demonstrate by immunoelectron microscope (IEM) [3]. MIWI starts its expression in the cytoplasm granules from P14 spermatocytes to the CB of round spermatids [19,22]; in this period, both MIWI and MILI bind an extremely abundant class of piRNA, the pachytene piRNA. Diversely, no matter expresses in prenatal PGCs or postnatal spermatocytes, each of these three PIWI proteins binds piRNA of a distinct size: MILI binds ~26 nt, MIWI2 interacts with ~28 nt whereas MIWI associates with ~29–31 nt [23,24].

Moreover, MIWI and MILI, but not MIWI2, bear RNA slicer activity essential for piRNA biogenesis and retrotransposon silencing [22,25]. Furthermore, several proteins are also crucial for the formation and maintaining of IMC and involved in the piRNA pathway, although they do not bind piRNA directly, these include mouse vasa homolog (MVH)/DDX4 [26,27,28], MITOPLD [29,30], and GASZ [31,32]. In the following sections we review and summarize the functions in male germ cell development of the proteins identified in IMC and CB during spermatogenesis from the current literature.

### 3.1. MVH (Mouse Vasa Homolog)/DDX4 

As a universal germ cell marker across the animal kingdom, MVH (vasa homolog gene) is expressed both in the process of spermatogenesis and oogenesis [26,33,34,35]. In testes, MVH is specifically expressed in germ cells from prenatal gonocytes at E10.5 to postnatal spermatogonium until round spermatids with the highest signal at the early stage of spermatocytes [26,33]. MVH displays a uniform expression pattern in the cytoplasm of spermatogonia and leptotene spermatocytes. Nevertheless, it appears to localize in several minuscule granules in the cytoplasm of zygotene spermatocytes and 3–5 larger granules in the cytoplasm of pachytene to diplotene spermatocytes, which are in the neighborhood of nuclei, and then localize in the chromatoid body of round spermatid stage [34]. Interestingly, MVH expresses in cytoplasmic granules of different sizes, including MILI-TDRD1 containing IMC and MIWI2-MAEL containing piP-bodies [3,34]. Further immunoelectron microscopy (IEM) confirms its enrichment in IMC between clustered mitochondria of spermatocytes [33]. Thus, its localization to different types of nuage implies the related communication and collaboration between them.

Male mice carrying homozygous mutations for *Mvh* gene are infertile, whereas homozygous female mutant mice are fertile with normal ovary histology. Detail histological examination of mice demonstrates that spermatogenesis arrested at the leptotene-zygotene stage and ceased differentiation into pachytene spermatocytes leading to a complete absence of post-meiotic germ cells upon *Mvh* deficiency [26]. In *Mvh* deficient fetal germ cells, the amount of piRNA significantly decreased mainly due to the failure of piRNA loading to MIWI2 despite the fact that MVH does not bind piRNA directly, while MILI-bound piRNA does not change, suggesting *Mvh* is needed between the first step of secondary piRNA production cleaved by MILI and piRNA loading to MIWI2 in the ping-pong cycle [3,26]. In addition, de novo methylation of retrotransposon severely decreased upon *Mvh* deficiency similar to that of *Mili*- and *Miwi2*-deficient mice [27,36].

Appealingly, MVH regulates the intracellular localization of several nuage proteins, including piP-body component MIWI2, IMC component TDRD1, and TDRD6. MIWI2 cannot locate in the nuclei and form granules in the cytoplasm but distributes uniformly in the cytoplasm, while the piP-body protein TDRD9 is still present as a granule in the cytoplasm, implying that MIWI2 cannot be recruited to piP-bodies probably due to the decreased piRNA loading [27]. Furthermore, the granular signals of MILI disappeared owing to the absence of the IMC structure in *Mvh* mutant cells. Meanwhile, IMC was also abolished, and the number of MVH-positive foci was significantly reduced in *Mvh* deficient germ cells examined by electron microscopy [27]. Similarly, TDRD1 exhibits diffuse distribution in the cytoplasm of *Mvh* mutant spermatogonia and spermatocytes rather than a granular signal in wild-type control. Contrarily, MVH expression is not altered in *Tdrd1* deficiency spermatocytes and spermatids [15,27]. In addition, TDRD1, TDRD6, and TDRD7 are physically associated with MVH protein and colocalize in the nuage of spermatocytes. Importantly, the co-localization of all three TDRD proteins at nuage is abolished upon *Mvh* mutation, which suggests that the MVH is essential for proper TDRD protein distribution and assembly in the nuage [37].

### 3.2. MILI

MILI expression is detected in both sexes as early as E12.5, when migrating PGCs reach the somatic genital ridge, and persists in both male and female germ cells after birth [20,38,39]. In adult testes, it expresses in the cytoplasm of spermatocytes and CB of round spermatids through spermatogenesis [24,39]. The numerous perinuclear granules of MILI signals were identified as enriched at IMC by immune-gold labeling in E18.5 gonocyte and colocalize with TDRD1, which is a known IMC component [3,24].

Similar to *Mvh* mutant mice, only male *Mili*-deficient mice are infertile. The testis weight of *Mili* mutant mice starts to decrease from two weeks after birth, suggesting normal PGC development and spermatogonia growth [20,40]. After that, *Mili*-deficient testis cease to grow, and no post-meiotic germ cells present in the adult testes. Spermatogenesis in *Mili* mutant mice arrests at the early stages of meiosis, probably at the zygotene or early pachytene stage of meiotic prophase [20]. Since MILI interacts with MVH and TDRD1, the cellular distribution of MVH and TDRD1 is detected, and MVH expression shows no change, but TDRD1 exhibited dramatically decreases in *Mili*-deficient spermatocytes [20,40].

Interestingly, the MILI expression was not affected by *Tdrd1* deficiency [20,40]. Additionally, loss of MILI in mice leads to aberrant localization of MIWI2, and complete loss of distribution of MAEL to piP-bodies and nuclei in gonocytes [41]. Simultaneously, the *Mili* mutants even display abnormal TDRD9 distribution (piP-body component) from both nuage and gonocyte nuclei, suggesting intense communication between IMC and piP-body and that MILI might collaborate with TDRD1 as the upstream of regulation of piP-body proteins [3,41].

### 3.3. MIWI

Unlike MILI and MIWI2, which begin their expression in fetal gonocytes, MIWI starts its expression in pachytene spermatocytes in the postnatal testes [19,39]. The mRNA of *Miwi* is first detected at P12 when zygotene spermatocytes appear, and its protein is first detectable at P14, corresponding to the pachytene spermatocyte stage. In adult seminiferous tubules, it enriches in the nuage of pachytene spermatocytes and CB of spermatids [22,39]. *Miwi* knockout male mice are infertile due to spermatogenesis block at round spermatid stage, and no spermatids go beyond step 4 [19]. As a bona fide RNA-guided RNase (slicer), its slicer activity is required for spermatogenesis because MIWI slicer activity-deficient mutant *Miwi^-/ADH^* male mice, which bear a point mutation D663A at the catalytic motif, are also infertile. Similar to the phenotype of *Miwi^-/-^* males, spermatogenesis arrested after meiosis at haploid round spermatids, as well as the disrupted structure of the chromatoid body in *Miwi^-/ADH^* mice [22].

Since MIWI and MILI interact with the IMC component TDRD1 in testes, the subcellular distribution of TDRD1 and MILI is mislocalized from the CB to diffuse expression in the cytoplasm and nuclei of round spermatids, probably due to the fuzzy chromatoid body structure in MIWI-deficient cells. By contrast, the IMC showed no obvious abnormality in *Miwi* mutant spermatocytes, and the localization of MILI and TDRD1 was normal [42].

In addition, MIWI directly binds with many specific piRNA-targeted mRNAs to regulate their degradation via its slicer activity, the expression level of the targeting mRNAs elevated in both *Miwi^-/-^* and MIWI slicer activity-deficient mutant *(Miwi^-/ADH^*) mice, which suggests the essential role of MIWI slicer activity in regulating spermatogenesis in a post-transcriptional manner [43].

### 3.4. Tudor domain-containing 1/Mouse Tudor repeat-1 (TDRD1/MTR-1)

Tudor domain-containing 1/Mouse Tudor repeat-1 (*Tdrd1*/*Mtr-1*) encodes four repeated copies of the Tudor domain and a single zinc-finger MYND domain [15,37]. It expressed in differentiating germ cells and predominantly localized to IMC in spermatogonia and spermatocytes and CBs in round spermatids [15,44]. A targeted mutation of the *Tdrd1* gene in mice resulted in complete male infertility, whereas mutant females were fertile [15]. *Tdrd1* mutant male seminiferous epithelium showed decreased pachytene spermatocytes and a lack of elongated spermatids and spermatozoa. Notably, the IMC in *Tdrd1* mutant spermatocytes was almost absent. In contrast, the CBs were observed in *Tdrd1* mutant spermatids, indicating that the origin of the CB might be different from IMC, or at least not totally the same as IMC [15].

Interestingly, in *Mili* mutant germ cells, the distribution of TDRD1 in the cytoplasm is dramatically reduced in both spermatogonia and spermatocytes owing to the decreased *Tdrd1* mRNA level; thus, there is significant protein reduction, implying that MILI is required for the expression or stability of TDRD1 at the mRNA level [40]. Curiously, given that both TDRD1 and MILI mutant male mice showed corruption of IMC structure and similar phenotype without post-meiotic spermatids, they might collaborate to recruit proper nuage components for the formation and functions of these structures, which are essential for the storage, metabolism, and transport of mRNA and small RNA during spermatogenesis.

Additionally, in *Tdrd1* mutant embryos (E18.5), piRNA significantly decreased due to the selective loss of MIWI2-bound piRNA populations; consistently, deletion of *Tdrd1* leads to a strong activation of LINE1 (long interspersed nuclear element 1) expression but not IAP (intra-cisternal A particle), indicating that TDRD1 plays an essential role for efficient ping-pong amplification cycle [41].

Together, these published data imply a pivotal role of TDRD1 in the regulation of piRNA pathway and IMC formation, as well as the effect on other piRNA pathway components such as the localization of MIWI2, providing substantial evidence for compartmentalization.

### 3.5. TDRD6

*Tdrd6* encodes seven Tudor domains (~250 KDa). It is abundant in the testes and predominantly localizes to IMC in spermatocytes and CBs in round spermatids as revealed by immunoelectron microscopy [37]. In detail, TDRD6 protein signal appears first in P17.5 primary spermatocytes which coincides with late pachytene, shown as multiple fine filamentous cytoplasmic granules; in P20.5 and P22.5 spermatids, TDRD6 appears as bright and distinct perinuclear dots in CB [16], which is consistent with the previously reported localization by immunoelectron microscopy [37].

Different from TDRD1, which localizes in fetal spermatogonia and female oocyte, TDRD6 does not express in spermatogonia and female oocyte [16]. Interestingly, TDRD6 has a C-terminal truncated protein (~230 KDa), which is only present in secondary spermatocytes and haploid spermatids, suggesting the C-terminal of TDRD6 has a function in a stage-specific manner during meiosis [16]. In addition, the full-length TDRD6 is preferentially localized in IMC, whereas the C-terminal truncated TDRD6 is highly enriched in the CB [16]. This unique expression pattern of TDRD6 is indicative of the dynamic change of protein composition, and thereby the biological function from IMC to CB.

*Tdrd6*-deficient male mice are infertile, whereas the females are fertile. The spermatogenesis of *Tdrd6* mutant mice blocked at the round spermatid stage [16]. The critical component of the CBs mislocalized, including MVH/DDX4, MIWI, and MAEL, and the ultrastructure of the CB under electron microscopy showed a diffuse, less condensed, and disrupted appearance. However, the structure of the IMC was not affected, suggesting that the influence of TDRD6 on IMC is still unclear [16].

Moreover, TDRD6 interacts with UPF1 and UPF2, which are vital proteins in the nonsense-mediated mRNA decay (NMD) pathway. Upon the removal of TDRD6, accompanied by distortion of CB, the interaction between MVH-UPF1 and UPF1-UPF2 was almost entirely lost, and UPF1 failed to co-localize with the CB. In contrast, the location of UPF2 was not affected, which disrupted the association of long 3’-UTR (3’-untranslated region) mRNA with UPF1 and UPF2, leading to increased mRNA stability and enhanced translational activity [45,46]. In sum, these studies suggest that TDRD6, as a component of IMC and CB, may play an essential role in germ cell development through mRNA regulation processes.

### 3.6. GASZ

Mouse *Gasz* is identified as a germ cell-specific gene, encoding a protein containing four ankyrin repeats (ANKs), a sterile-α motif (SAM), and a leucine zipper domain, also termed as ASZ1 and evolutionarily conserved between species [47,48]. GASZ expresses in both male and female germ cells, which shows the weak granular signals in the cytoplasm of spermatogonia and pre-leptotene spermatocytes, the highest expression in mid-late pachytene spermatocytes until round spermatids. Notably, whether GASZ localizes mitochondria outer membrane depends on its C-terminal mitochondrial targeting sequences and is mainly co-expressed with IMC component MILI both in the gonocytes and spermatocytes, and partially co-localizes with MVH and TDRD1, indicating its enrichment in IMC. In contrast, in round spermatid, GASZ is still expressed as a granular signal in the cytoplasm partially co-staining with mitochondria, but not localized in the CB [31,46,49]. 

Like all other IMC component genes, *Gasz* knockout males are sterile, and mutant females are fertile. Adult *Gasz* mutant males exhibit a striking diminishment of spermatocytes, no post-meiotic spermatids, and spermatozoa, which is phenocopied by the zygotene-pachytene spermatocyte block observed in MILI-, MVH-, and MIWI2-null males [31]. Interestingly, MILI, MVH, and TDRD1 mislocalized in *Gasz* mutant germ cells. Thereinto, MILI shows the most significant change, which is mostly disappeared in gonocytes to utterly absent in the newborn spermatogonia; similarly but less significantly, MVH and TDRD1 display reduced perinuclear expression but diffuse distribution in the cytoplasm both in E16.5 gonocytes and newborn spermatogonia [31]. Moreover, GASZ interacts with several IMC components, including MIWI, MVH, TDRD1, and RANBP9, but not MILI and MAEL, confirming GASZ is one member of the IMC network [31]. Meanwhile, many nuage proteins were reduced in GASZ null testes, including MIWI, TDRD1, MVH, MILI, TDRD6, and TDRD7, and the IMC structure was absent in GASZ-null germ cells, implying the essential role of GASZ in the formation and/or maintenance of IMC and the correct localization of nuage proteins [31]. Likewise, retrotransposons were markedly hypomethylated and activated, including Line1 and intra-cisternal A particle (IAP) in *Gasz* mutant newborn testes, similar to *Mili*- and *Miwi2*-null testes [31]. 

Of note, the mitochondrial localization sequence (MLS) of GASZ is essential for GASZ to regulate spermatogenesis because the MLS deletion GASZ mutants (*Gasz*^ΔNLS/ΔNLS^) almost display the same phenotype with *Gasz*^-/-^ mice, including male infertility but not female, diffused localization of IMC component, absent IMC, and activated transposons [32]. It is probably through the interaction with the outer membrane mitochondrial proteins (MFN1 and MFN2), thereby regulating mitochondrial fusion [32,50].

### 3.7. Moloney leukemia virus 10 like 1 (MOV10L1)

*Mov10l1* (Moloney leukemia virus 10 like 1) is a testis-specific homologous in mice and humans to the putative RNA helicase *Armitage* gene in Drosophila and implicated in miRNA and piRNA pathways [51,52,53,54]. In postnatal testis, MOV10L1 is most strongly expressed in the cytoplasm of pachytene spermatocyte, but weakly in newborn gonocytes, and absent in post-meiotic round spermatids. The global deletion of MOV10L1 RNA helicase domain results in male infertility and spermatogenesis blocked at the zygotene-pachytene stage [55]. Moreover, MOV10L1 interacts with MILI, MIWI, MIWI2, and TDRD1 but not MVH, MILI, and TDRD1 [55,56]. Markedly, retrotransposons Line1 and IAP activate due to demethylation upon MOV10L1 deletion. Furthermore, MIWI2 mislocalizes from the nuclei to the cytoplasm, and both MILI and MIWI2-bound piRNA are devoid in newborn *Mov10l1*^-/-^ testes. Therefore, the RNA helicase domain of MOV10L1 is essential for the localization of some nuage proteins and the biogenesis and/or stability of both prenatal and pre-pachytene piRNAs [55,56].

In addition to its involvement in pre-pachytene piRNA biogenesis, MOV10L1 also takes part in the biogenesis of pachytene piRNA by supporting the unloading of pachytene piRNA to MIWI in *Neurog3*-Cre- and *Hspa2*-Cre-mediated conditional knockout of *Mov10l1* [57,58]. Likewise, both *Neurog3*-Cre- and *Hspa2*-Cre-induced *Mov10l1* knockout males are infertile due to spermiogenesis arrest at the round spermatids stage at step 4 and step 8, respectively. Significantly, MIWI-bound piRNAs are undetectable in *Neurog3*-Cre-*Mov10l1* knockout testes and dramatically decreased in *Hspa2*-Cre-*Mov10l1* knockout testes, probably owing to piRNA biogenesis defects considering the accumulation of pachytene piRNA precursors in *Mov10l1* mutant testes. Consequently, mitochondria form a single large polar cluster in *Mov10l1* null pachytene spermatocytes similar to the *MitoPLD* mutant spermatocytes, accompanied by a congregation of IMC components, including MILI, MIWI, TDRD1, and GASZ. Taken together, MOV10L1 might function as a master regulator in the upstream of PIWI proteins to regulate piRNA biogenesis and contribute to IMC formation in mice [59]. 

### 3.8. TDRKH/TDRD2

TDRKH, a Tudor and KH domain-containing protein, attracts attention as a PIWI-interacting protein, including MIWI and MIWI2, but not MILI in testis, via its Tudor domain to identify symmetrically demethylated arginines in the N-terminus of MIWI and MIWI2 [11,60]. Immunoelectron microscopy revealed that TDRKH co-localizes with mitochondria and IMC in pro-spermatogonia, spermatogonia, and spermatocytes [11]. 

*Tdrkh* global knockout females are fertile, but males are sterile due to meiosis arrest at the zygotene stage [11]. Furthermore, the mislocalization of nuage proteins was observed upon *Tdrkh* deletion, including IMC component TDRD1 and piP-body protein MIWI2 with the most severe change, but not MILI and MVH [11]. TDRD1 lost its occupation in IMC, and MIWI2 lost its distribution in the nuclei and piP-body in E18.5 *Tdrkh* mutant prospermatogonia. Nevertheless, the IMC structure remains normally present, implying that *Tdrkh* is indispensable for the formation of IMC but might provide a platform to recruit nuage proteins to locate correctly [11]. Meanwhile, LINE1 dramatically upregulated in *Tdrkh* mutant testes due to CpG hypomethylation, similar to the results in piRNA pathway mutants, such as MIWI, MILI, and MOV10L1. Importantly, both prenatal piRNA (E18.5) and pre-pachytene piRNA (P17) are almost absent in *Tdrkh* mutant testis due to the piRNA 3’-end trimming defects of piRNA precursors,1 whereas the secondary piRNA biogenesis is intact [11,61]. Thus, *Tdrkh* is the first identified piRNA trimming factor, which functions in the primary piRNA biogenesis but not the secondary piRNA biogenesis pathway. 

Inspiringly, the conditional knockout of *Tdrkh* mediated by *Stra8*-Cre (*Tdrkh* ^cKO^) leads to male infertility owing to spermatogenesis block at step 5 of round spermatids [62]. MILI-bound piRNAs dramatically decreased in abundance without trimming; meanwhile, MIWI-bound piRNAs were completely lost because the MIWI cannot be recruited to the IMC in *Tdrkh*^cKO^ spermatocytes. Furthermore, the structure of the CB is disrupted, and the localization of MIWI in the CB is lost but not for MILI and MVH in *Tdrkh* ^cKO^ round spermatids [62]. Mechanically, since *Tdrkh* itself does not contain any nuclease domain, its 3’-trimming might work through recruitment and cooperation with trimmer PNLDC1, which is pivotal for mammalian piRNA 3′-trimming and is essential for transposon silencing and spermatogenesis in mice [63,64,65].

### 3.9. MITOPLD

MITOPLD (also known as PLD6) is the only ortholog of Drosophila Zuc in humans, which is conserved in vertebrates, including zebrafish, frogs, mice, rats, and humans and involved in primary piRNA biogenesis [29]. In mice, MITOPLD is highly expressed in testis and growing oocytes, and its expression in testis is from E16.5 to adult stage in both mRNA and protein levels. Based on its mitochondria-targeted signal in the N-terminal region, mouse MITOPLD is enriched in the mitochondrial outer membranes in fetal and adult mice testes [29]. *Mitopld* global knockout female mice are fertile, but males are infertile owing to defects in spermatocytes at P16 when differentiation reaches mid-pachytene spermatocyte. The spermatogenesis of *Mitopld* mutant mice is blocked at the zygotene stage, resulting in complete loss of spermatids in adult seminiferous tubules. Furthermore, *Mitopld* deficient spermatogonia display derepression of retrotransposons and disrupted piRNA biogenesis both in fetal and postnatal testes [29].

Notably, MITOPLD deficiency results in the mislocalization of the piRNA pathway components and mitochondria. In E16.5 *Mitopld* mutant prospermatogonia, the IMC proteins (MILI and TDRD1) together with piP-body protein (MIWI2) exhibit crescent-shaped staining adjacent to the nuclei rather than granular signals in the cytoplasm of wild-type controls, which is similar with their abnormal expression in *Mvh* mutant prospermatogonia [29]. However, the distribution of p-body protein DDX6 is normal in *Mitopld* mutant cells. Furthermore, mitochondria accumulate at one side, and the IMC is absent in *Mitopld* mutant prospermatogonia [29,30]. Thus, MITOPLD is essential for the distribution of mitochondria, the formation of IMC and targeting of nuage proteins to different nuage structures, and MITOPLD and MVH have a similar regulation role for piRNA components.

MITOPLD is localized at the outer mitochondrial membrane and its phospholipase activity hydrolyzes a mitochondria-specific lipid cardiolipin to produce phosphatidic acid (PA), which is a pleiotropic and rapidly metabolized signaling lipid and function as a membrane anchor to regulate mitochondrial fusion and morphology [66], implying the link between the lipid metabolism of mitochondria in piRNA biogenesis. Noteworthily, the deletion of the enzyme *Lipin1*, which hydrolyzes mitochondrial phosphatidic acid, also results in male infertility and gives rise to the opposite effect to *Mitopld* mutant, displaying increased density of IMC in *Lipin1* mutant germ cells [30]. Together with the fact that MITOPLD deficiency in male mice leads to piRNA elimination, transposon activation, spermatogenesis arrest, and IMC disappearance, we thus conclude that although whether MITOPLD enriched in IMC needs to be further confirmed, the above findings demonstrate its requirement for the organization and sustainment of IMC, and the proper concentration of phosphatidic acid. It might provide a balance to regulate the formation of IMC and assembly of IMC components around mitochondria.

### 3.10. DDX25/GRTH

DDX25 (also named as gonadotropin-regulated testicular RNA helicase, GRTH) is a testis-specific member of DEAD (Glu-Asp-Ala-Glu)-box protein family, which is well-known to participate in RNA metabolism and translational processes [67]. Immunoelectron microscopy revealed that DDX25 enriches in several compartments of male germ cells, including but not restricted to, IMC of spermatocytes and CB in round spermatids [68]. DDX25 knockout female mice are fertile, but males are sterile owing to spermatogenesis arrest at step 8 of round spermatids and failure to elongate [69]. The structure of CB is dramatically condensed and reduced in size throughout all steps of DDX25 mutant round spermatids, causing ceased spermiogenesis [69]. MVH and MIWI lost their localization in CB of *Ddx25* mutant round spermatids [70].

In male germ cells, DDX25 localizes in the cytoplasm and nucleus of spermatocytes and round spermatids, and two DDX25 protein species are present, including a 56 KDa non-phosphorylated form enriched in the nucleus and a 61 KDa phosphorylated form located in the cytoplasm [71]. The non-phosphorylated DDX25 interacts with chromosome region maintenance-1 protein (CRM1), an evolutionarily highly conserved protein, and is essential for nuclear RNP (ribonucleoprotein) particles export [72] to participate in the nuclear export pathway for mRNA transport. Whereas the cytoplasmic DDX25 is associated with actively translating polyribosomes and is believed to regulate mRNA translation, it binds with selective mRNA messages whose proteins express at different steps of spermiogenesis, including *Tnp1, Tnp2, Prm1,* and *Prm2*. Importantly, the protein levels of PGK2 (phosphoglycerate kinase 2), tACE (testicular converting enzyme), and TNP2 are absent in purified DDX25 mutant round spermatids without RNA level change, implying DDX25 might be a post-transcriptional regulator [69,71].

Importantly, different polymorphisms of the *Ddx25* gene are reported in infertile Chinese and Japanese men, respectively. A silent mutation (Cys) located in exon 10 (c.852 G→T) was the most relevant mutation identified in infertile men with idiopathic azoospermia or oligozoospermia from west China [73]. In infertile Japanese men, a missense mutation Arg^242^His in exon 8 was identified, which leads to the absence of the phosphorylated form of DDX25 [74]. Interestingly, the knock-in male mice of Arg^242^His mutation (DDX25-KI^h/h^) are sterile due to spermatogenesis block at step 8 of round spermatids and a substantial decrease in size of CBs, which are in line with the phenotypes of knockout mice. Further, a complete absence of the 61 KDa phosphorylated DDX25, chromatin remodeling, and related proteins such as TNP2, PRM2, and TSSK6, and their reduced mRNA half-lives in DDX25-KI^h/h^ support its role in mRNA stability and translation [75]. Thus, the feature of IMC localization for DDX25 is essential for its function in spermatogenesis and male fertility.

### 3.11. Other Intermitochondrial Cement (IMC) and Chromatoid Body (CB) Proteins

In addition to the above IMC proteins, immunoelectron microscopy also revealed the IMC and CB localization of MAEL, AGO2, and DDX6. However, it is noteworthy that these proteins are not restricted to IMC and CB, and they enriched in other nuage structures as well, including piP-body and satellite body [76,77,78]. MAEL located in IMC of spermatocytes and CB of round spermatids co-stains with DDX4, MIWI, and DDX25 [77]. *Mael* knockout male mice are infertile due to meiosis arrest, LINE1 derepression at the onset of meiosis, decreased piRNAs, massive DNA damage, as well as defective homologous chromosome synapsis [79,80], which are reminiscent of the phenotypes observed in *Mili* and *Miwi2* mutants [20,21]. AGO2 is an essential component for the processing of small interfering RNA (siRNA)-directed RNA interference (RNAi) in the RNA-induced silencing complex (RISC) [76]. Similarly, dual immunoelectron microscopy staining revealed that AGO2 was also located in the IMC of spermatocytes and CB of round spermatids and co-localized with nuage protein MAEL and MIWI [76]. DDX6 is expressed in both the cytoplasm and nucleus of spermatogenic cells and is strongly enriched in IMC of pachytene spermatocytes and weakest in CB of round spermatids [78].

## 4. The Communication between IMC (pi-bodies) and piP-Bodies for piRNA Biogenesis and Spermatogenesis

### 4.1. Multiple Localization

Several proteins are enriched but not restricted to IMC, such as MVH, TDRKH, MAEL, and DDX6, which localize in several different germinal granules, including piP-body and P-body, implying their functional complexity and specificity as described above [3,11,33,34,78,79]. Interestingly, most IMC proteins interact with MVH, which is localized in both IMC and piP-body, as summarized in Table 1. In addition, the deletion of some IMC components impact on not only the IMC proteins but also the piP-body proteins, such as GASZ; the knockout of *Gasz* leads to the mislocalization of MIWI, TDRD1, MVH, MILI, TDRD6, and TDRD7 [31]. However, the deletion of several piP-body proteins has no impact on the location and expression of IMC components; as an example take piP-body protein, GTSF1, whose deletion leads to loss of MIWI2-bound piRNAs and male infertility [81,82,83]. GTSF1 foci completely co-localize with MIWI2-TDRD9 foci but partially co-stain with MILI signal and interact with both MILI and MIWI2. In addition, piP-body components mislocalize in Gtsf1-deficient prospermatogonia, including MAEL, MIWI2, and TDRD9, but the localization of IMC components are unaffected including MVH, MILI, and TDRD1 [81], further supporting the interplay between IMC and piP-body.

### 4.2. Primary piRNA Processing

The majority of piRNAs transcribed from RNA polymerase II transcription units named piRNA cluster in the nucleus. Following nuclear export, piRNA precursors are processed by cytoplasmic proteins, mainly located in nuage. There are two piRNA biogenesis pathways, including the primary processing pathway and the ping-pong amplification cycle. In the first pathway, the single-stranded piRNA precursors are cleaved into thousands of non-overlapping/phased fragments by processing machinery in the cytoplasm, and then the fragments are loaded into Piwi proteins where they transform into mature piRNAs with a strong preference for a 5’ uridine (U1 bias) [88,89,90]. Thereinto, the primary piRNA processing machinery involves multiple key factors, including nuage components such as MITOPLD, MOV10L1, MVH, and TDRKH. MOV10L1 specifically binds and unwinds piRNA precursors and generates piRNA precursor intermediate fragments (PPIFs), which initiate the first cleavage step of piRNA processing [84,91]. Meanwhile, the processing of piRNA precursors into piRNA intermediates is also regulated by the conserved mitochondrial outer membrane protein MITOPLD, which is essential for primary piRNA biogenesis but not secondary piRNA biogenesis [29]. Additionally, as a conserved mitochondrial Tudor domain protein located in IMC, piP-body and MAM structures, TDRKH plays a vital role in piRNA 3’ trimming via associating with the trimmer PNLDC1 during fetal piRNA biogenesis [63,64,65] and recruiting MIWI but not MILI to mitochondria in pachytene spermatocytes, therefore revealing that the mitochondria surface serves as a scaffolding for piRNA biogenesis [62].

### 4.3. Ping-Pong Amplification Cycle

Ping-pong amplification cycle couples piRNA biogenesis and transposon silencing occurs in fetal prospermatogonia/gonocytes, but not in adult spermatocytes [24,92]. In fetal prospermatogonia/gonocytes, ping-pong amplification cycle of piRNA biogenesis takes place between IMC and piP-body regulated by MILI and MIWI2, which work in a distinct but complementary way to generate piRNAs and methylate transposon sequences thereafter to repress transposable element (TE) expression [3,24]. In the ping-pong cycle during the development of prospermatogonia, MILI-combined primary sense piRNAs recognize and cleave the transcripts that orientated from antisense transposon sequences and generate secondary piRNAs, which then load the target-derived piRNAs into both MILI and MIWI2 complex. After cytoplasmic loading, MIWI2 translocates to the nucleus where it accomplishes its transcriptional silencing functions [3,93]. Therefore, MILI is in favor of primary piRNAs and 1U-containing piRNA produced in the ping-pong cycle, whereas MIWI2 mainly combined with secondary sequences.

Importantly, in the ping-pong cycle, both MILI and MIWI2 cooperate with other nuage proteins. MILI interacts with IMC component MVH/DDX4 and TDRD1, and both the mRNA and protein levels of TDRD1 are reduced in *Mili* mutant testis [40]. Although the localization of MVH/DDX4 is normal in *Mili* mutant, MVH/DDX4 is essential for the maturation of MILI cleaved pre-piRNA intermediates depending on its ATPase activity; thus by involving the ping-pong cycle, loss of ATPase activity leads to male infertility displaying piRNA intermediate accumulation and transposon activation [28]. Meanwhile, MIWI2 associates with TDRD9 and MAEL in mice; the localization of MAEL, MIWI2, and TDRD9 is dependent on MILI function, but not vice-versa. Further, the assembly of MIWI2 and TDRD9 onto piP-bodies is regulated by MAEL, indicating that MAEL acts downstream of MILI but upstream of MIWI2 and TDRD9 [24,41].

Interestingly, in *Mili*-deficient germ cells, MIWI2 could not locate in the nuclei but uniformly distributed in the cytoplasm. On the contrary, the MILI foci in the cytoplasm did not change in *Miwi2* mutant germ cells. Dramatically, MIWI2-bound piRNAs are absent upon MILI deficiency, implying MILI is essential for piRNA loading to MIWI2 and supporting the directionality of their relationship between MILI and MIWI2. MILI is responsible for triggering the ping-pong cycle with primary piRNAs, and MIWI2 binds with secondary piRNAs [24,93]. Thus, although IMC-located MILI and piP-body-located MIWI2 foci do not co-localize with each other, they are physically nearby, which is required for the ping-pong amplification cycle.

Altogether, both IMC and piP-body are distinct subcellular compartmentalizations where piRNA pathway proteins assemble in spermatogenesis. Regularly, IMC components mainly regulate the primary piRNA pathway, and piP-body proteins are predominantly involved in secondary piRNA amplification. IMC components play multiple distinct roles through interplay with other nuage proteins, including piRNA biogenesis pathway, mRNA storage, export and translation, alternative splicing, and DNA damage (Figure 1B). Importantly, germline mutations of several IMC components were identified in human patients with oligoasthenoteratozoospermia/azoospermia, such as PIWI (MIWI), DDX25, TDRD6, and MITOPLD [94]. In conclusion, the normal physical function of IMC and the communication between IMC and piP-body are essential for the sustainment of spermatogenesis.

## 5. MAMs in Spermatogenesis

Despite widespread recognition of the importance of MAMs in neurodegenerative diseases and cancer [6,7], the understanding of how MAMs modulate spermatogenesis is minimal. Our previous research reported the abundance of MAMs in both human and mouse testicular spermatocytes, and many piRNA pathway components exist in testicular MAMs revealed by quantitative proteomic analyses, including MIWI, MILI, GASZ, and TDRKH [8]. However, only very few proteins are confirmed by Western blot in testis, such as TDRKH and DAZL (deleted in azoospermia-like) [8,11]. In the following sections, we summarize the functions of proteins as MAM components identified from our previous study in testes.

### 5.1. TDRKH

As an IMC component, TDRKH also co-localizes with mitochondria and is enriched at MAMs [11]. Its mutants exhibit meiotic arrest at the zygotene stage, accompanied by hypomethylation of *Line1* DNA, upregulated *Line1* mRNA, and protein, pre-pachytene, and pachytene piRNA trimming defects [11,62]. *Tdrkh*-deleted testis fails to recruit MIWI but not MILI to the mitochondrial membrane, leading to disrupted MIWI-piRNAs and MILI-piRNA trimming errors as described above.

### 5.2. DAZL

DAZL (deleted in azoospermia-like) is a germ cell restricted RNA binding protein, and its expression is a hallmark of vertebrate germ cells [95,96,97]. Together with DAZ and BOULE, they consist of a human fertility family, which is necessary for gametogenesis in worms, flies, mice, and humans [98]. DAZL co-localizes with MIWI in pachytene spermatocytes and exhibits higher expression levels in the cytoplasm of pachytene spermatocytes [85,86]. The global knockout of *the Dazl* gene leads to severe disruption of testicular histology with a nearly complete loss of germ cells beyond the spermatogonial stage [85]. DAZL can regulate mRNA translation by binding to poly(A) RNAs and cooperation with poly(A) binding protein (PABP) and cytoplasmic polyadenylation element-binding protein (CPEB) [87,99,100,101].

*Dazl* predominantly functions directly as a positive post-transcriptional regulator through 3’-UTR interactions via the motif UGUU, thereby controlling a network of specific genes required for germ cell survival [86,102]. Stage-specific deletion of *Dazl* in gonocytes (*Vasa*-Cre mediated, VKO), spermatogonia (*Stra8*-Cre mediated, SKO), and spermatocytes (*Hspa2*-Cre mediated, HKO) all result in complete male sterility [86]. Characterization by an absence of germ cells in VKO due to a significant decrease of spermatogonia-associated DAZL target protein expression, zygotene stage arrest in SKO due to the disrupted assembly of the synaptonemal complex and DNA repair, and round spermatid stage arrest in HKO mice, confirm its requirement at these three stages of spermatogenesis by recruiting PABP to target DAZL mRNAs to regulate their translation [86].

## 6. Concluding Remarks

Different from the non-membrane cloudy structure between mitochondria and IMC, MAMs are mitochondria-associated ER membranes, which form a narrow apace between mitochondria and the ER as an exchange channel for calcium and lipids in male germ cells. Despite the structural divergence between IMC and MAMs, the similarities between them include the involvement of mitochondrial outer membranes. Thus, it provides the possibility that some IMC proteins also localize in MAMs during spermatogenesis, especially the mitochondrial outer membrane proteins, such as TDRKH, which might serve as a mitochondrial scaffold to recruit piRNA pathway factors [62]. Interestingly, mammalian oocytes also contain MAM structure [103,104], but no reports demonstrate that female germ cells have IMC and CB-like structures during oogenesis. Yet, the functions and underlying mechanism of MAM formation in both male and female germ cells are still elusive to date.

IMC and MAM components participate in multiple biological functions in spermatogenesis, including piRNA biogenesis, DNA damage repair, mRNA export, and translation. Within nuage, RNA must be directed into specific proteins to maintain continuous piRNA ping-pong looping. Since piRNA biogenesis occurs in the cytoplasm involving several nucleases enriched in IMC, such as MIWI and MILI, outer mitochondrial membrane-anchored proteins might cooperate with IMC components to accomplish piRNA biogenesis pathway. However, the functional relationship between IMC and MAMs still needs to be further explored.

Provokingly, mitochondria are scattered in spermatocytes and round spermatids but aligned in the middle piece of elongating spermatids and spermatozoa forming a mitochondrial capsule (MC). The mitochondrial capsule (MC) is maintained by connecting mitochondria with outer-dense fibers (ODFs) regulated by GOPC, GPX4 and KLC3, and SPATA19 prevents dispersal of mitochondria as reviewed recently [105]. As a structural component of the mitochondrial capsule, GPX4 constitutes at least 50% of the mitochondrial capsule material and harbors three isoforms—cytosolic, mitochondrial, and nuclear-encoded—by the same gene [106,107,108]. Both the conditional knockout of *Gpx4* in spermatocytes and the deletion of its mitochondrial form of *Gpx4* (mGpx4) lead to male infertility due to severe structural defects of sperm with irregular mitochondrial alignment and swollen mitochondria [109,110]. *Spata19* null mice are infertile from sperm motility defects with the improper mitochondrial organization and reduced ATP production, indicating its role in the organization of mitochondria capsule [111]. However, when and how the mitochondrial capsule (MC) forms and whether there is a relationship between IMC, chromatoid body (CB), and the mitochondrial capsule (MC) is still an enigma.

This study considers the dynamic morphology of mitochondria in different types of germ cells during spermatogenesis, thereby meeting the different energy needs in different cell types [112,113]. The location of several IMC and MAM components is also dynamic, with expression in different granules, raising the possibility that the components of IMC may serve a function in MAMs. Despite the significant barriers to investigating the direct functions of IMC and MAMs in the male germline, it is worthy of exploring whether the morphological change of mitochondria influences the location and function of IMC and MAM proteins in spermatogenesis in the future.

In sum, this review highlights the mitochondria-associated germinal structures and summarizes the functions of the components during spermatogenesis and piRNA biogenesis (Figure 1A,B), which leads us to endeavor a new research direction in the male reproductive field.

## Figures and Tables

**Table 1:** The components of mitochondria-associated germinal structures in murine testes are shown.

Name	Localization	Functions	Mutant Phenotype	Block Stage	Interaction Proteins	References
MVH	IMC, piP-body, CB	piRNA biogenesis	Male infertile; Female fertile	Leptotene-zygotene stage; IMC undetectable	TDRD1, TDRD2 (TDRKH), TDRD6, MIWI, MILI, RANBP9	[3,26,27,28,33,34]
MILI	IMC, CB	piRNA biogenesis; mRNA translation	Male infertile; Female fertile	Zygotene-pachytene stage; IMC undetectable	MVH/DDX4, TDRD1, TDRD2, GTSF1	[3,20,24,40,81]
MIWI	IMC, CB	piRNA biogenesis; mRNA translation	Male infertile	Step 4 early round spermatid stage; Normal IMC, but fuzzy CB	TDRD1, TDRD2 (TDRKH), TDRD6,	[22,39,42]
TDRD1/MTR1	IMC, CB	piRNA biogenesis	Male infertile; Female fertile	Pachytene-diplotene stage IMC reduction; CB damaged	MVH/DDX4, MIWI, MILI, MIWI2	[15,41,44]
TDRD6	IMC, CB	miRNA expression, nonsense mediated mRNA decay (NMD), spliceosome maturation and mRNA splicing	Male infertile; Female fertile	Round spermatid stage; IMC not detected; Disrupted CB	MVH/DDX4, MIWI, MILI, UPF1, UPF2, PRMT5, SmB	[16,37,45,46]
GASZ	IMC, mitochondria	piRNA metabolism	Male infertile; Female fertile	Zygotene-pachytene block; IMC disappeared	MIWI, TDRD1, MVH, MFN1, MFN2, DAZL RANBP9,	[31,32,47,48,49,50]
MOV10L1	IMC, mitochondria	piRNA metabolism	Male infertile; Female fertile	Zygotene-pachytene block; Mitochondria accumulation, disrupted CB	MILI, MIWI, MIWI2, TDRD1, HSPA2	[55,56,84]
TDRKH/TDRD2	IMC, p-body, piP-body, MAMs, mitochondria	piRNA trimming	Male infertile; Female fertile	Zygotene-pachytene block	MIWI, MIWI2	[11,60,61,62]
MITOPLD	IMC, mitochondria	Primary piRNA biogenesis; Mitochondria shape	Male infertile; Female fertile	Zygotene stage; Absent IMC	Lipin 1b	[29,30]
DDX25	IMC	mRNA export and translation regulation	Male infertile; Female fertile	Step 8 of round spermatids, condensed and reduced CB	Chromosome region maintenance-1 protein (CRM1),	[68,69,70,71,74]
DAZL	MAMs, cytoplasm	mRNA translation	Male infertile	Absence of germ cells in *Vasa*-Cre; Zygotene stage arrest in *Stra8*-Cre; Round spermatid stage arrest in *Hspa2*-Cre	PABP, CPEB	[85,86,87]

Abbreviations: MVH, mouse vasa homolog; MILI, piwi-like RNA-mediated gene silencing 2; MIWI, piwi-like RNA-mediated gene silencing 1; TDRD1/MTR1, Tudor domain-containing 1/Mouse Tudor repeat-1; IMC, intermitochondrial cement; CB, chromatoid body; MAMs, mitochondria-associated ER membranes; GASZ, ankyrin repeat, SAM and basic leucine zipper domain containing 1; MOV10L1, Moloney leukemia virus 10 like 1; TDRKH, Tudor and KH domain-containing protein; MITOLPD, phospholipase D family member 6; PABP, poly(A) binding protein; UPF1, UPF1 regulator of nonsense transcripts homolog; HSPA2, heat shock protein 2; CPRB, cytoplasmic polyadenylation element-binding protein; RANBP9, RNA binding protein 9; GTSF1, gametocyte specific factor 1; DAZL, deleted in azoospermia-like.
